# The Geropathology Grading Platform demonstrates that mice null for Cu/Zn-superoxide dismutase show accelerated biological aging

**DOI:** 10.1007/s11357-018-0008-0

**Published:** 2018-02-24

**Authors:** Timothy A. Snider, Arlan Richardson, Julie A. Stoner, Sathyaseelan S. Deepa

**Affiliations:** 1 Department of Veterinary Pathobiology, Center for Veterinary Health Sciences, Oklahoma State University, Stillwater, OK USA; 2 Department of Geriatric Medicine/Reynolds Oklahoma Center on Aging, University of Oklahoma Health Sciences Center, Oklahoma City, OK USA; 3 Oklahoma City VA Medical Center, Oklahoma City, OK USA; 4 Department of Biostatistics and Epidemiology, University of Oklahoma Health Sciences Center, Oklahoma City, OK USA

**Keywords:** Pathology, Cu/Zn-superoxide dismutase, Aging, Geropathology Grading Platform, Healthspan

## Abstract

The Geropathology Grading Platform (GGP) that is being developed by the Geropathology Research Network provides a grading system that allows investigators to assess biological aging in mice by measuring the pathological status of a wide range of tissues in a standardized scoring system. The GGP is a grading system that generates a numerical score for the total lesions in each tissue, which when averaged over the mice in the cohort provides a composite lesion score (CLS) for each tissue and mouse. In this study, we tested ability of the GGP to predict accelerated aging in mice null for Cu/Zn-superoxide dismutase (Sod1KO mice), which have been shown to have reduced lifespan and healthspan. Using the GGP, we evaluated the pathological status of 11 tissues from male and female wild-type (WT) and Sod1KO mice at 9 to 10 months of age. The whole animal CLS was 2- to 3.5-fold higher for both male and female Sod1KO mice compared to WT mice. The tissues most affected in the Sod1KO mice were the liver, lung, and kidney. These data demonstrate that the GGP is able to predict the accelerated aging phenotype observed in the Sod1KO mice and correlates with the changes in healthspan that have been reported for Sod1KO mice. Thus, the GGP is a new paradigm for evaluating the effect of an intervention on the pathological status of an animal as well as the healthspan of the mice.

**Electronic supplementary material:**

The online version of this article (10.1007/s11357-018-0008-0) contains supplementary material, which is available to authorized users.

## Introduction

It is widely acknowledged that pathological information is an important component of studies focused on aging. For example, lifespan data without pathological data limits the information one can gain about aging (Bronson 1993). This limitation becomes critical when interventions of any sort are studied because it is difficult to conclude from survival data alone if changes in lifespan arise because aging has been altered by the experimental manipulation, e.g., is a broad spectrum of disease processes or pathological lesions modified, which is predicted if aging has been altered or if the change in lifespan arise because only one or two disease processes or pathological lesions are altered. Currently, most pathological analyses of aging colonies of rodents have focused on identifying the number of mice with specific pathological lesions and in some cases scoring the severity of each lesion. While an increase in pathology is a hallmark of aging, the types of lesions that occur with age and the tissues in which they occur can vary greatly from animal to animal making it necessary to study a large number of animals to be able to statistically determine the effect of aging or an intervention on each lesion.

A Geropathology Grading Platform (GGP) was recently developed by the Geropathology Grading Committee chaired by Dr. Warren Ladiges and composed of six board-certified veterinary pathologists (Ladiges et al. 2016, 2017; Ladiges 2017). The Geropathology Grading Committee was part of the NIA funded Geropathology Research Network that developed a grading system allowing investigators to assess biological aging in mice by measuring the pathological status of a wide range of tissues in old mice. The GGP is based on a standardized set of guidelines developed by the Geropathology Grading Committee to (1) detect the presence or absence of low-impact histopathological lesions that occur with age and (2) determine the level of severity of high-impact lesions in tissues of aged mice. The GGP allows one to generate a numerical score for each lesion in a tissue that is then summed to give a score for the total lesions in the tissue that is then averaged for the mice in the cohort to obtain a composite lesion score (CLS) for that tissue. In other words, the CLS gives one a numerical value for the burden of lesions, which can be compared to similar values from other mice of other cohorts to measure changes with age or with an aging intervention. Ladiges et al. (2017) showed that the CLS for the heart, lung, kidney, and liver increased dramatically with age in two strains of mice, C57BL/6N and CB6F1 mice. They also reported that rapamycin treatment, which has been shown to increase the lifespan of mice and maintain or improve various measures of function (Richardson, 2013; Johnson et al., 2013), reduced the CLS in multiple tissues. Thus, the GGP is a new paradigm for measuring the pathology associated with aging that allows investigators to potentially assess biological aging in mice.

The focus of this study was to test the ability of the GGP to predict accelerated aging using mice null for Cu/Zn-superoxide dismutase (Sod1KO mice). Cu/Zn-superoxide dismutase is the major superoxide dismutase isozyme that catalyzes the conversion of superoxide anions to hydrogen peroxide, and Sod1KO mice show high levels of oxidative stress in various tissues and plasma (Muller et al., 2006). In 2005, Huang’s laboratory reported that Sod1KO mice show a ~30% decrease in lifespan (Elchuri et al., 2005). Our group confirmed that the lifespan of Sod1KO mice was reduced, e.g., median lifespan was reduced from ~30 months for wild-type (WT) mice to ~22 months for Sod1KO mice (Zhang et al., 2013). In addition to exhibiting a decrease in lifespan, the Sod1KO mice exhibited various accelerated aging phenotypes, e.g., hearing loss (Keithley et al., 2005), cataracts (Olofsson et al. 2007), skin thinning and delayed wound healing (Iuchi et al. 2010) and loss of muscle mass (Muller et al. 2006). The Sod1KO mice also exhibit reduced physiological functions compared to WT mice, e.g., reduced grip strength, rota-rod performance, spontaneous wheel running activity, and endurance exercise capacity (Deepa et al., 2017). Using the GGP, we show in this study that the CLS for adult Sod1KO mice was significantly higher than the CLS for adult WT mice.

## Methods

The Sod1KO mice (C57BL/6J background) used in this study were generated by Drs. Charles Epstein and Ting-Ting Huang and were genotyped as previously described (Elchuri et al. 2005). The mice were fed a standard NIH-31 chow and maintained under barrier conditions in micro-isolator cages on a 12-h dark/light cycle. In this study, male and female mice at 9 to 10 months of age were studied, 8 WT and 8 Sod1KO male mice and 6 WT and 10 Sod1KO female mice. For tissue collection, animals were sacrificed by CO_2_ inhalation followed by cervical dislocation, and the tissues were immediately excised and immersion-fixed in 10% buffered neutral formalin. Following appropriate fixation of approximately 1 week, tissues were processed routinely, e.g., paraffin embedded, sectioned via microtomy at 4 μm, stained with haemotoxylin and eosin, and coverslipped. Slides were labeled by mouse number and group number, but identifying details of groups were blinded to the scoring pathologist. All procedures were approved by the Institutional Animal Care and Use Committee at the University of Oklahoma Health Sciences Center and the Oklahoma City VA Medical Center.

### Pathological analysis

All mice examined and scored possessed a full complement of organs subjected to observation and scoring, and these included the heart, lung, liver, kidney, spleen, pancreas, small intestine, stomach, salivary gland, reproductive tissues appropriate to gender, mesenteric lymph node, and spinal cord. In a few rare cases, there was not a full complement of tissues and these cases are designated by an NP (Not Present) in Supplementary Table 1. Organs were scored as visualized on H&E-stained slides via light microscopy by an ACVP board-certified pathologist. The pathologist was blinded to the identities of the experimental groups.

A pre-determined repertoire of scoreable lesions was assessed and scored in each organ; this repertoire has been determined by the Geropathology Grading Committee. Each score is recorded in Supplementary Table 1. We note and emphasize that the Geropathology Grading Committee developing the Geropathology Grading Platform is actively preparing a full descriptive methodology (manuscript in preparation). In this study, we used the following scoring system: significant lesions within organs are scored 0–3 (0 = absent, 1 = mild, 2 = moderate, 3 = severe) and insignificant or incidental lesions are scored 0–1 (0 = absent, 1 = present) or 0–2 (0 = absent, 1 = present in rare foci, 2 = present in multiple foci). A full description of the scoreable parameters, typical lesions and grades seen in each organ, and pictorial atlas correlates goes beyond the scope of this methodological description; however, a full methodological description is available via personal communication to the corresponding author or via Dr. Warren Ladiges, chair of the Geropathology Grading Committee.

### Statistical analysis

Lesion scores were entered into a spreadsheet systematically by mouse, by tissue, and by scored lesion (see Supplementary Table 1). The numerical scores for the lesions in each tissue were then summed to give a score for the total lesions in the tissue that is then averaged to give the CLS for each tissue and each mouse. The means and standard errors of the mean are then calculated across the cohorts, and the data statistically analyzed using a two-tailed Student’s *t* tests. The Spearman’s rank correlation coefficient (Daniel & Cross, 2014) was used determine the strength of the linear association of the CLSs between pairs of tissues. This analysis was used because the bivariate distribution of the lesion numbers is not normally distributed. A value of 1 indicates a perfect positive linear association (more lesions in one tissue correlated with more lesions in another tissue). A value of 0 indicates no linear association and a value of − 1 indicates a perfect negative linear association (more lesions in one tissue corresponds to fewer lesions in another tissue). Cronbach’s alpha, which ranges from 0 to 1 where a value of 1 corresponds to perfect internal consistency among the measures, was then calculated to evaluate the overall degree of correlation among the number of lesions among liver, kidney, and lung tissues (Cronbach, 1951). A 2-sided 0.05 alpha level was used to define statistical significance.

## Results

**Fig. 1:**
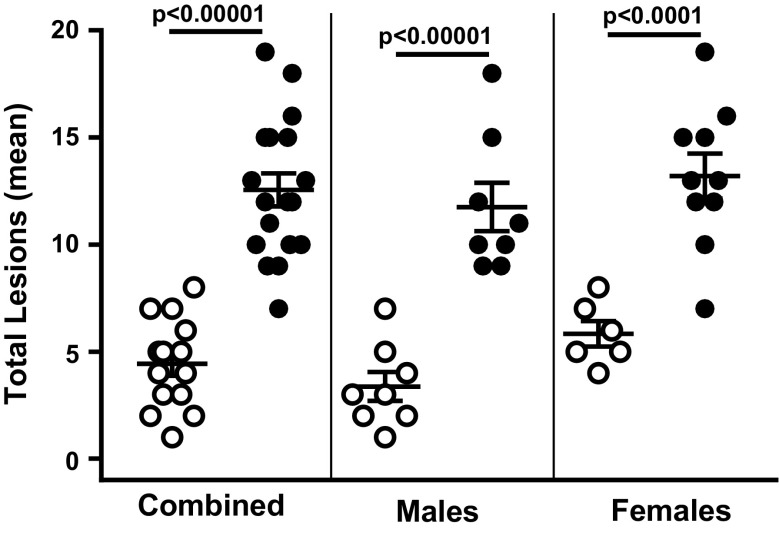
The whole animal composite lesion scores for WT and Sod1KO mice. The graphs show the CLSs for all 11 tissues for each WT (open circles) and Sod1KO (closed circles) mouse. The mean and SEM are shown for 6 to 10 of mice in each group: combined (male and female), male, and female mice. *P* values are based on a two-sided independent sample *t* test

In this study, we observed and recorded the lesion scores of 9- to 10-month-old male and female WT and Sod1KO mice. The lesion scores for each of the mice for each of 11 tissues we examined are given in the Supplementary Table 1. We first summed the lesion scores for all 11 tissues for each of the four cohorts of mice to obtain a composite lesion score (CLS) for the whole animal. Figure 1 shows the whole animal CLS for the male and female WT and Sod1KO mice. Even at a relatively young age, i.e., 9 to 10 months of age, we observed a dramatic (2- to 3.5-fold) increase in the whole animal CLS for both male and female Sod1KO mice compared to WT mice.

**Fig. 2:**
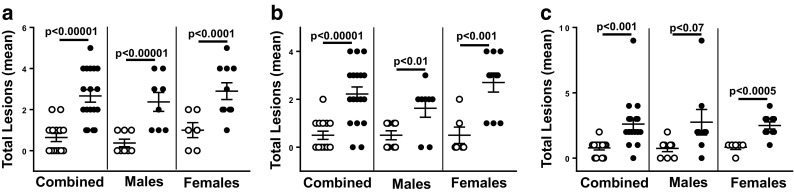
Composite lesion scores for liver, lung, and kidney from WT and Sod1KO mice. The graphs show the CLSs for the liver (**a**), lung (**b**), and kidney (**c**) for each WT (open circles) and Sod1KO (closed circles) mouse. The mean and SEM are shown for 6 to 10 of mice in each group: combined (male and female), male, and female mice. *P* values are based on a two-sided independent sample *t* test

We next assessed the CLS for each of the 11 tissues, and these data are given in Table 1. It is clear that the tissues most affected by knocking out the *Sod1* gene were the liver, lung, and kidney. Figure 2 shows the graphical representation of the CLSs for these three tissues. The CLSs we observed for the liver, lung, and kidney of male WT mice, which are on the C57BL/6J background, are similar to the CLSs that Ladiges et al. (2017) and Ladiges (2017) reported for these tissues from 8-month-old male C57BL/6N. The CLSs for the liver and lung for the Sod1KO and WT mice were similar to the CLS for all 11 tissues, i.e., the same conclusion could be drawn from assessing either of these tissues as measuring the CLS for all tissues. However, when one compares the CLS for all tissues (Fig. 1) to the CLSs for either the liver or lung (Fig. 2), it is evident that the differences between the Sod1KO and WT mice are more robust when comparing CLS for all 11 tissues than either the liver or lung.

**Table 1:** Composite lesion scores (CLSs) for wild-type (WT) and Sod1KO Mice for Each Tissue

Tissue	Genotype	Combined (AVG ± SEM)	Male (AVG ± SEM)	Female (AVG ± SEM)
Liver	WT	0.6 ± 0.2	0.4 ± 0.2	1.0 ± 0.4
	Sod1KO	2.6 ± 0.3 (*p* < 0.0001^*^)	2.4 ± 0.5 (*p* = 0.001)	2.9 ± 0.4 (*p* = 0.006)
Lung	WT	0.5 ± 0.2	0.5 ± 0.2	0.5 ± 0.3
	Sod1KO	2.2 ± 0.3 (*p* < 0.0001)	1.6 ± 0.4 (*p* = 0.02)	2.7 ± 0.4 (*p* = 0.002)
Kidney	WT	0.8 ± 0.2	0.8 ± 0.3	0.8 ± 0.2
	Sod1KO	2.6 ± 0.4 (*p* = 0.002)	2.8 ± 1.0 (*p* = 0.07, NS)	2.5 ± 0.3 (*p* = 0.0005)
Pancreas	WT	0.6 ± 0.3	0.8 ± 0.5	0.3 ± 0.2
	Sod1KO	1.0 ± 0.2 (NS)	1.0 ± 0.3 (NS)	1.0 ± 0.1 (*p* = 0.02)
Heart	WT	0.1 ± 0.1	0.1 ± 0.1	0
	Sod1KO	0.3 ± 0.1 (NS)	0.1 ± 0.1(NS)	0.4 ± 0.2 (NS)
Salivary gland	WT	0.6 ± 0.2	0.4 ± 0.2	1.0 0.3
	Sod1KO	1.4 ± 0.2 (*p* = 0.004)	1.4 ± 0.4 (*p* = 0.03)	1.5 ± 0.2 (NS)
Skin	WT	0	0	0
	Sod1KO	0.1 ± 0.1 (NS)	0.1 ± 0.1 (NS)	0 (NS)
Gastro intestinal	WT	0	0	0
	Sod1KO	0.1 ± 0.1 (NS)	0	0.1 ± 0.1 (NS)
Spinal cord	WT	0	0	0
	Sod1KO	0	0	0
Reproductive*	WT	ND	0.4 ± 0.2	0
	Sod1KO	ND	2.1 ± 0.4 (*p* = 0.0006)	0
Lymph node	WT	0	0	0
	Sod1KO	0	0	0

Sample sizes: 8 WT and 8 Sod1KO male mice and 6 WT and 10 Sod1KO female mice. ND: The CLS for males and females combined were not determined because of the differences in the reproductive systems

*All *P* values based on a two-tailed Student’s *t* test

These data are consistent with previous reports on end-of-life pathology of the Sod1KO mice. Both Elchuri et al. (2005) and Zhang et al. (2013) reported an increase in neoplastic lesions in the Sod1KO mice, especially hepatocellular carcinoma. In addition, Zhang et al. (2017) reported the incidence of renal pathology was greater in Sod1KO mice. It is interesting to note that the CLSs for these three tissues show a trend for a sex difference; female Sod1KO mice have higher CLSs for the liver, lung, and kidney than male Sod1KO mice; however, these differences were not significant. On the other hand, the CLS for the reproductive system of male Sod1KO was significantly higher (~fivefold) that WT male mice; however, the reproductive system of female Sod1KO mice did not show any evidence of lesions at 9 to 10 months of age.

To determine if Sod1KO mice that show a high CLS in one tissue show an increase in CLS in the other tissues, we used the Spearman’s rank correlation coefficient, which allows us to estimate the strength of the linear association among each pair of tissues. As Table 2 shows, the correlation coefficients are small in magnitude indicating a weak positive association, which is not statistically significant. The data in Table 2 also show that the associations between tissues tend to be greater in female Sod1KO mice.

**Table 2:** Comparison of composite lesion scores (CLSs) between tissues

Comparison	Combined	Females	Males
Liver-Lung	0.16 (*p* = 0.53)	0.23	− 0.14
Liver-Kidney	0.21 (*p* = 0.40)	0.45	0.00
Lung-Kidney	0.37 (*p* = 0.13)	0.31	0.36

The Spearman’s rank correlation coefficient is given for the comparison of the CLSs between the tissues shown. The *p* values (in parenthesis) are only shown for the combined males and females because the sample size for the males and females is too small to draw any conclusions. As an overall measure of the degree of correlation among the CLS measures, the Cronbach’s alpha was calculated to be 0.33, 0.56, and 0.18 for the combined, female, and male values, respectively. The Cronbach’s alpha values indicate a low level of correlation among all three measures for data from males and a low to moderate level of correlation for the combined groups and females

**Fig. 3:**
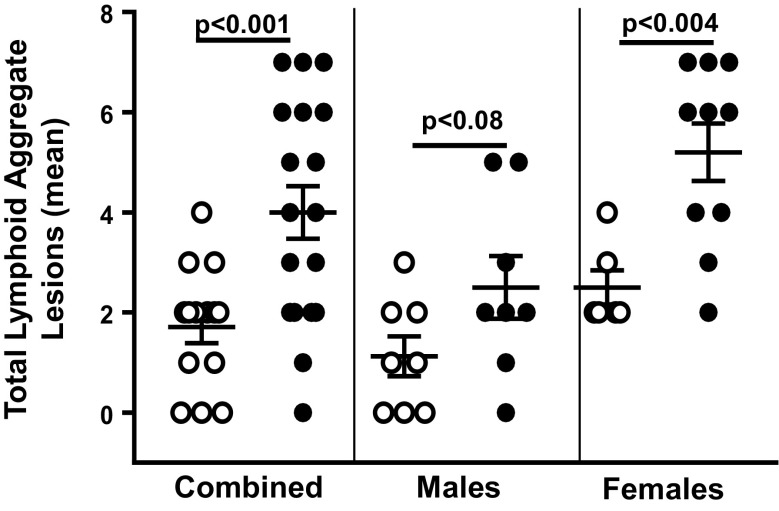
Total lymphoid aggregate lesions for WT and Sod1KO mice. The graphs show the total number of lymphoid aggregate lesions in the 11 tissues for each WT (open circles) and Sod1KO (closed circles) mouse. The mean and SEM are shown for 6 to 10 of mice in each group: combined (male and female), male, and female mice. *P* values are based on a two-sided independent sample *t* test

The GGP also allows one to measure similar lesions across tissues, e.g., we observed lymphoid aggregate lesions in many of the tissues. Therefore, we measured the lymphoid aggregate scores for the four cohorts of mice across the 11 tissues. Lymphoid aggregates are defined as aggregated, nodular clusters of well-differentiated lymphocytes and fewer plasma cells, usually in foci unaffected by other obvious lesions. Though it is uncertain whether these aggregates have any functional consequence, it is common to observe an increase in these aggregates with chronological age. The data in Fig. 3 show that female Sod1KO mice have a greater level of lymphoid aggregate lesions compared to WT mice. Although the male Sod1KO mice show an increase in lymphoid aggregates, the increase is not significant.

## Discussion

Lifespan has been the gold standard used to assess the ability of an intervention to modulate aging. However, the consensus of the aging research community over the past decade is that it is important to show that a manipulation not only increases lifespan but also improves/maintains functional outcomes, e.g., healthspan. Assessing the healthspan of an animal, such as mouse, is neither straight forward nor easy. For example, rapamycin, which has been consistently shown to increase the lifespan of mice (Richardson et al., 2015) gives mixed results when a large number of physiological parameters are measured (Richardson, 2013; Johnson et al., 2013). While many aging phenotypes are restored (e.g., behavior/cognition, several immune parameters, and a number of pathological lesions), many functions were not significantly altered. The interpretations of such “segmental” effects demonstrate the difficulty in determining if a manipulation improves healthspan. For example, must all processes that change with age need to be reversed or improved? And, if not, what percent of the functional measures have to be enhanced/improved for an intervention to be considered as improving healthspan? Or are some functions, e.g., cognition, immune function, and cardiac/muscle function, more important than others in assessing the impact of a manipulation on healthspan? The problems faced in assessing healthspan were recently demonstrated by Fischer et al. (2016) when they measured several physiological functions in the same mice to determine if all physiological functions were altered similarly with age in each mouse, i.e., did mice with reduced function by one measure of physiological function show reduced function in other measures of physiological function. They found there were few correlations among the various functional assays in the old mice, indicating that function in the various physiological domains changed with age independent of one another. They also failed to find any of the measures of physiological function that were significantly associated with an increased probability of premature death in the old mice.

An increase in pathology is a hallmark of aging, occurring in all living organisms in most, if not all tissues. Because the greater the pathology burden of an animal is more likely correlated to a reduced functional status of the animal, a global pathological assessment of an animal is a potentially novel way to indirectly assess the functional status of an animal. Thus, manipulations that reduce or delay the age-related increase in pathology would be predicted to lead to improved function, i.e., healthspan. In contrast, manipulations that decrease lifespan and reduce physiological functions would be predicted to lead to increased pathology. Using the GGP developed by the Geropathology Grading Committee, we compared the pathological status of Sod1KO and WT mice. It should be noted that the Sod1KO mice show ~30% decrease in lifespan. The median lifespan of Sod1KO mice was 674 vs 913 days for wild-type mice. The maximum life span, as measured by the 90th percentile survival, of Sod1KO mice was 862 vs 1054 days for wild-type mice (Elchuri et al., 2005; Zhang et al., 2013). It should be noted that while the Sod1KO mice show ~30% decrease in lifespan, we measured the pathological status of the mice at 9 to 10 months of age when less than 10% of the animals have died. When we summed the lesions over all 11 tissues analyzed, we found that the whole animal CLS was dramatically increased (2- to 3.5-fold) in Sod1KO mice compared to WT mice. This increase was observed for both male and female mice. The Sod1KO mice show an accelerated aging phenotype as measured by increased oxidative damage (Muller et al., 2006), mitochondria dysfunction (Jang et al., 2010), and increased cell senescence and inflammation (Zhang et al., 2017). More importantly, the Sod1KO mice at 9 to 10 months of age show evidence of reduced healthspan as measured by physiological deficits in grip strength, rota-rod performance, wheel running, and endurance exercise (Deepa et al., 2017). Thus, the whole animal CLS mirrors the changes in the measures of healthspan that have been observed in the Sod1KO mice.

In summary, our data show that the GGP developed by the Geropathology Grading Committee detected a dramatic increase in pathology in 9- to 10-month-old Sod1KO mice, which is consistent with the accelerated aging phenotype previously reported for Sod1KO mice. These data combined with the data from Ladiges et al. (2017), which showed that the CLS for tissues from two strains of mice increased dramatically with age, demonstrate that the GGP is a new paradigm for measuring the pathology associated with aging. Our data also suggest that that GGP can be used to assess the healthspan of mice because the increase in the CLS for the whole animal mirrors the decline in physiological functions that have been observed in Sod1KO mice. An obvious limitation in using GGP to assess the pathological/health status of mice is that it is a terminal assay; therefore, it cannot be used as part of a longitudinal study to predict the outcomes of individual mice.

## Electronic supplementary material

Supplementary Table 1 (DOCX 144 kb)
