# Supplementary material for: The Geropathology Grading Platform demonstrates that mice null for Cu/Zn-superoxide dismutase show accelerated biological aging
Source: GeroScience. 2018 Feb 24;40(2):97–103. doi: 10.1007/s11357-018-0008-0 (PMC5964058; doi:10.1007/s11357-018-0008-0)
Supplement: Supplementary file 1 — (DOCX 144 kb) [file 11357_2018_8_MOESM1_ESM.docx]

**Supplementary Table 1. The lesion scores for wild type (WT) and Sod1KO mice for each of eleven tissues**

**Liver**

| Sex | Strain | HepDeg | HepFib | HepLip | HepAtr | PortInf | BilHyp | LymAgg | uGran | Tumor | Amyloid | EMH | Total |
| --- | --- | --- | --- | --- | --- | --- | --- | --- | --- | --- | --- | --- | --- |
| Male | WT-1 | 0 | 0 | 0 | 0 | 0 | 0 | 0 | 0 | 0 | 0 | 0 | 0 |
|  | WT-2 | 0 | 0 | 0 | 0 | 0 | 0 | 0 | 0 | 0 | 0 | 0 | 0 |
|  | WT-3 | 1 | 0 | 0 | 0 | 0 | 0 | 0 | 0 | 0 | 0 | 0 | 1 |
|  | WT-4 | 0 | 0 | 0 | 0 | 0 | 0 | 0 | 0 | 0 | 0 | 0 | 0 |
|  | WT-5 | 1 | 0 | 0 | 0 | 0 | 0 | 0 | 0 | 0 | 0 | 0 | 1 |
|  | WT-6 | 1 | 0 | 0 | 0 | 0 | 0 | 0 | 0 | 0 | 0 | 0 | 1 |
|  | WT-7 | 0 | 0 | 0 | 0 | 0 | 0 | 0 | 0 | 0 | 0 | 0 | 0 |
|  | WT-8 | 0 | 0 | 0 | 0 | 0 | 0 | 0 | 0 | 0 | 0 | 0 | 0 |
|  | Sod1KO-1 | 2 | 0 | 1 | 0 | 0 | 0 | 1 | 0 | 0 | 0 | 0 | 4 |
|  | Sod1KO-2 | 2 | 0 | 1 | 0 | 1 | 0 | 0 | 0 | 0 | 0 | 0 | 4 |
|  | Sod1KO-3 | 2 | 0 | 0 | 0 | 0 | 0 | 0 | 0 | 0 | 0 | 0 | 2 |
|  | Sod1KO-4 | 1 | 0 | 0 | 0 | 0 | 0 | 0 | 0 | 0 | 0 | 0 | 1 |
|  | Sod1KO-5 | 2 | 0 | 1 | 0 | 0 | 0 | 0 | 0 | 0 | 0 | 0 | 3 |
|  | Sod1KO-6 | 2 | 0 | 1 | 0 | 0 | 0 | 0 | 0 | 0 | 0 | 0 | 3 |
|  | Sod1KO-7 | 1 | 0 | 0 | 0 | 0 | 0 | 0 | 0 | 0 | 0 | 0 | 1 |
|  | Sod1KO-8 | 1 | 0 | 0 | 0 | 0 | 0 | 0 | 0 | 0 | 0 | 0 | 1 |
| Female | WT-1 | 0 | 0 | 0 | 0 | 0 | 0 | 0 | 0 | 0 | 0 | 0 | 0 |
|  | WT-2 | 1 | 0 | 0 | 0 | 0 | 0 | 1 | 0 | 0 | 0 | 0 | 2 |
|  | WT-3 | 0 | 0 | 0 | 0 | 0 | 0 | 0 | 0 | 0 | 0 | 0 | 0 |
|  | WT-4 | 0 | 0 | 0 | 0 | 0 | 0 | 1 | 0 | 0 | 0 | 0 | 1 |
|  | WT-5 | 0 | 0 | 0 | 0 | 0 | 0 | 1 | 0 | 0 | 0 | 1 | 2 |
|  | WT-6 | 0 | 0 | 0 | 0 | 0 | 0 | 0 | 1 | 0 | 0 | 0 | 1 |
|  | Sod1KO-1 | 1 | 0 | 0 | 0 | 0 | 0 | 0 | 0 | 0 | 0 | 1 | 2 |
|  | Sod1KO-2 | 2 | 0 | 0 | 0 | 0 | 0 | 0 | 1 | 0 | 0 | 1 | 4 |
|  | Sod1KO-3 | 1 | 0 | 0 | 0 | 0 | 0 | 0 | 0 | 0 | 0 | 0 | 1 |
|  | Sod1KO-4 | 1 | 0 | 1 | 0 | 0 | 0 | 0 | 1 | 0 | 0 | 0 | 3 |
|  | Sod1KO-5 | 0 | 0 | 0 | 0 | 0 | 0 | 0 | 1 | 0 | 0 | 1 | 2 |
|  | Sod1KO-6 | 2 | 0 | 0 | 0 | 0 | 0 | 0 | 1 | 1 | 0 | 1 | 5 |
|  | Sod1KO-7 | 1 | 0 | 0 | 0 | 0 | 0 | 0 | 0 | 0 | 0 | 1 | 2 |
|  | Sod1KO-8 | 1 | 0 | 0 | 0 | 0 | 0 | 1 | 1 | 0 | 0 | 1 | 4 |
|  | Sod1KO-9 | 1 | 0 | 0 | 0 | 0 | 2 | 0 | 0 | 0 | 0 | 1 | 4 |
|  | Sod1KO-10 | 1 | 0 | 0 | 0 | 0 | 0 | 1 | 0 | 0 | 0 | 0 | 2 |

**Kidney**

| Sex | Strain | GN | TuDeg | Min | Inf | Hnep | LyAgg | Tum | Amy | Total |
| --- | --- | --- | --- | --- | --- | --- | --- | --- | --- | --- |
| Male | WT-1 | 0 | 1 | 0 | 0 | 0 | 0 | 0 | 0 | 1 |
|  | WT-2 | 0 | 1 | 0 | 0 | 0 | 1 | 0 | 0 | 2 |
|  | WT-3 | 0 | 1 | 0 | 0 | 0 | 0 | 0 | 0 | 1 |
|  | WT-4 | 0 | 1 | 0 | 0 | 0 | 0 | 0 | 0 | 1 |
|  | WT-5 | 0 | 0 | 0 | 0 | 0 | 0 | 0 | 0 | 0 |
|  | WT-6 | 0 | 0 | 0 | 0 | 0 | 1 | 0 | 0 | 1 |
|  | WT-7 | 0 | 0 | 0 | 0 | 0 | 0 | 0 | 0 | 0 |
|  | WT-8 | 0 | 0 | 0 | 0 | 0 | 0 | 0 | 0 | 0 |
|  | Sod1KO-1 | 0 | 1 | 0 | 0 | 0 | 1 | 0 | 0 | 2 |
|  | Sod1KO-2 | 0 | 1 | 0 | 0 | 0 | 1 | 0 | 0 | 2 |
|  | Sod1KO-3 | 0 | 1 | 0 | 0 | 0 | 1 | 0 | 0 | 2 |
|  | Sod1KO-4 | 1 | 1 | 1 | 0 | 0 | 0 | 1 | 0 | 4 |
|  | Sod1KO-5 | 0 | 0 | 0 | 0 | 0 | 0 | 0 | 0 | 0 |
|  | Sod1KO-6 | 3 | 3 | 1 | 1 | 0 | 1 | 0 | 0 | 9 |
|  | Sod1KO-7 | 1 | 0 | 0 | 0 | 0 | 1 | 0 | 0 | 2 |
|  | Sod1KO-8 | 0 | 0 | 0 | 0 | 0 | 1 | 0 | 0 | 1 |
| Female | WT-1 | 0 | 0 | 0 | 0 | 0 | 1 | 0 | 0 | 1 |
|  | WT-2 | 0 | 0 | 0 | 0 | 0 | 1 | 0 | 0 | 1 |
|  | WT-3 | 0 | 0 | 0 | 0 | 0 | 1 | 0 | 0 | 1 |
|  | WT-4 | 0 | 0 | 0 | 0 | 0 | 0 | 0 | 0 | 0 |
|  | WT-5 | 0 | 0 | 0 | 0 | 0 | 1 | 0 | 0 | 1 |
|  | WT-6 | 0 | 1 | 0 | 0 | 0 | 0 | 0 | 0 | 1 |
|  | Sod1KO-1 | 0 | 0 | 0 | 0 | 0 | 1 | 0 | 0 | 1 |
|  | Sod1KO-2 | 1 | 0 | 1 | 0 | 0 | 2 | 0 | 0 | 4 |
|  | Sod1KO-3 | 1 | 0 | 0 | 0 | 0 | 1 | 0 | 0 | 2 |
|  | Sod1KO-4 | 1 | 0 | 0 | 0 | 0 | 1 | 0 | 0 | 2 |
|  | Sod1KO-5 | 1 | 1 | 0 | 0 | 0 | 1 | 0 | 0 | 3 |
|  | Sod1KO-6 | 1 | 1 | 0 | 0 | 0 | 1 | 0 | 0 | 3 |
|  | Sod1KO-7 | 0 | 1 | 0 | 0 | 0 | 1 | 0 | 0 | 2 |
|  | Sod1KO-8 | 1 | 1 | 0 | 0 | 0 | 1 | 0 | 0 | 3 |
|  | Sod1KO-9 | 1 | 1 | 0 | 0 | 0 | 0 | 0 | 0 | 2 |
|  | Sod1KO-10 | 1 | 1 | 0 | 0 | 0 | 1 | 0 | 0 | 3 |

**Heart**

| Sex | Strain | ArtScl | AthScl | M Fib | CMP | M it is | V-Myx | V-Fib | LyAgg | Min | AtrThr | Tum | Amy | Total |
| --- | --- | --- | --- | --- | --- | --- | --- | --- | --- | --- | --- | --- | --- | --- |
| Male | WT-1 | 0 | 0 | 0 | 0 | 0 | 0 | 0 | 0 | 0 | 0 | 0 | 0 | 0 |
|  | WT-2 | 0 | 0 | 0 | 0 | 0 | 0 | 0 | 0 | 0 | 0 | 0 | 0 | 0 |
|  | WT-3 | 0 | 0 | 0 | 0 | 0 | 0 | 0 | 1 | 0 | 0 | 0 | 0 | 1 |
|  | WT-4 | 0 | 0 | 0 | 0 | 0 | 0 | 0 | 0 | 0 | 0 | 0 | 0 | 0 |
|  | WT-5 | 0 | 0 | 0 | 0 | 0 | 0 | 0 | 0 | 0 | 0 | 0 | 0 | 0 |
|  | WT-6 | 0 | 0 | 0 | 0 | 0 | 0 | 0 | 0 | 0 | 0 | 0 | 0 | 0 |
|  | WT-7 | 0 | 0 | 0 | 0 | 0 | 0 | 0 | 0 | 0 | 0 | 0 | 0 | 0 |
|  | WT-8 | 0 | 0 | 0 | 0 | 0 | 0 | 0 | 0 | 0 | 0 | 0 | 0 | 0 |
|  | Sod1KO-1 | 0 | 0 | 0 | 0 | 0 | 0 | 0 | 0 | 0 | 0 | 0 | 0 | 0 |
|  | Sod1KO-2 | 0 | 0 | 0 | 0 | 0 | 0 | 0 | 0 | 0 | 0 | 0 | 0 | 0 |
|  | Sod1KO-3 | 0 | 0 | 0 | 0 | 0 | 0 | 0 | 0 | 0 | 0 | 0 | 0 | 0 |
|  | Sod1KO-4 | 0 | 0 | 0 | 0 | 0 | 0 | 0 | 0 | 0 | 0 | 0 | 0 | 0 |
|  | Sod1KO-5 | 0 | 0 | 0 | 0 | 0 | 0 | 0 | 0 | 0 | 0 | 0 | 0 | 0 |
|  | Sod1KO-6 | 0 | 0 | 0 | 0 | 0 | NP | NP | 0 | 0 | 0 | 0 | 0 | 0 |
|  | Sod1KO-7 | 0 | 0 | 1 | 0 | 0 | NP | NP | 0 | 0 | 0 | 0 | 0 | 1 |
|  | Sod1KO-8 | 0 | 0 | 0 | 0 | 0 | NP | NP | 0 | 0 | 0 | 0 | 0 | 0 |
| Female | WT-1 | 0 | 0 | 0 | 0 | 0 | NP | NP | 0 | 0 | 0 | 0 | 0 | 0 |
|  | WT-2 | 0 | 0 | 0 | 0 | 0 | NP | NP | 0 | 0 | 0 | 0 | 0 | 0 |
|  | WT-3 | 0 | 0 | 0 | 0 | 0 | NP | NP | 0 | 0 | 0 | 0 | 0 | 0 |
|  | WT-4 | 0 | 0 | 0 | 0 | 0 | NP | NP | 0 | 0 | 0 | 0 | 0 | 0 |
|  | WT-5 | 0 | 0 | 0 | 0 | 0 | NP | NP | 0 | 0 | 0 | 0 | 0 | 0 |
|  | WT-6 | 0 | 0 | 0 | 0 | 0 | NP | NP | 0 | 0 | 0 | 0 | 0 | 0 |
|  | Sod1KO-1 | 0 | 0 | 1 | 0 | 0 | NP | NP | 0 | 0 | 0 | 0 | 0 | 1 |
|  | Sod1KO-2 | 0 | 0 | 0 | 0 | 0 | NP | NP | 0 | 0 | 0 | 0 | 0 | 0 |
|  | Sod1KO-3 | 0 | 0 | 0 | 0 | 0 | NP | NP | 0 | 0 | 0 | 0 | 0 | 0 |
|  | Sod1KO-4 | 0 | 0 | 0 | 0 | 0 | NP | NP | 0 | 0 | 0 | 0 | 0 | 0 |
|  | Sod1KO-5 | 0 | 0 | 0 | 0 | 0 | 0 | 0 | 0 | 0 | 0 | 0 | 0 | 0 |
|  | Sod1KO-6 | 0 | 0 | 0 | 0 | 0 | 0 | 0 | 1 | 0 | 0 | 0 | 0 | 1 |
|  | Sod1KO-7 | 0 | 0 | 0 | 0 | 1 | NP | NP | 1 | 0 | 0 | 0 | 0 | 2 |
|  | Sod1KO-8 | 0 | 0 | 0 | 0 | 0 | NP | NP | 0 | 0 | 0 | 0 | 0 | 0 |
|  | Sod1KO-9 | 0 | 0 | 0 | 0 | 0 | NP | NP | 0 | 0 | 0 | 0 | 0 | 0 |
|  | Sod1KO-10 | 0 | 0 | 0 | 0 | 0 | NP | NP | 0 | 0 | 0 | 0 | 0 | 0 |

**Lung**

| Sex | Strain | AMP | AlHis | IntPn | Airway | RespHyp | VasHyp | Pl-it is | PulFib | LyAgg | Tum | Total |
| --- | --- | --- | --- | --- | --- | --- | --- | --- | --- | --- | --- | --- |
| Male | WT-1 | 0 | 0 | 0 | 0 | 0 | 0 | 0 | 0 | 0 | 0 | 0 |
|  | WT-2 | 0 | 0 | 0 | 0 | 0 | 0 | 0 | 0 | 0 | 0 | 0 |
|  | WT-3 | 0 | 0 | 0 | 0 | 0 | 0 | 0 | 0 | 1 | 0 | 1 |
|  | WT-4 | 0 | 0 | 0 | 0 | 0 | 0 | 0 | 0 | 0 | 0 | 0 |
|  | WT-5 | 0 | 0 | 0 | 0 | 0 | 0 | 0 | 0 | 1 | 0 | 1 |
|  | WT-6 | 0 | 0 | 0 | 0 | 0 | 0 | 0 | 0 | 1 | 0 | 1 |
|  | WT-7 | 0 | 0 | 0 | 0 | 0 | 0 | 0 | 0 | 1 | 0 | 1 |
|  | WT-8 | 0 | 0 | 0 | 0 | 0 | 0 | 0 | 0 | 0 | 0 | 0 |
|  | Sod1KO-1 | 0 | 1 | 0 | 0 | 0 | 0 | 0 | 0 | 2 | 0 | 3 |
|  | Sod1KO-2 | NP | NP | NP | NP | NP | NP | NP | NP | NP | NP | 0 |
|  | Sod1KO-3 | 0 | 1 | 0 | 0 | 0 | 0 | 0 | 0 | 1 | 0 | 2 |
|  | Sod1KO-4 | 0 | 1 | 0 | 0 | 0 | 0 | 0 | 1 | 0 | 0 | 2 |
|  | Sod1KO-5 | 0 | 0 | 0 | 0 | 0 | 0 | 0 | 0 | 0 | 0 | 0 |
|  | Sod1KO-6 | 0 | 1 | 0 | 0 | 0 | 0 | 0 | 0 | 1 | 0 | 2 |
|  | Sod1KO-7 | 0 | 0 | 0 | 0 | 1 | 0 | 0 | 0 | 1 | 0 | 2 |
|  | Sod1KO-8 | 0 | 0 | 0 | 0 | 1 | 0 | 0 | 0 | 1 | 0 | 2 |
| Female | WT-1 | 0 | 0 | 0 | 0 | 0 | 0 | 0 | 0 | 0 | 0 | 0 |
|  | WT-2 | 0 | 0 | 0 | 0 | 0 | 0 | 0 | 0 | 0 | 0 | 0 |
|  | WT-3 | 0 | 1 | 1 | 0 | 0 | 0 | 0 | 0 | 0 | 0 | 2 |
|  | WT-4 | 0 | 0 | 0 | 0 | 0 | 0 | 0 | 0 | 0 | 0 | 0 |
|  | WT-5 | 0 | 0 | 0 | 0 | 0 | 0 | 0 | 1 | 0 | 0 | 1 |
|  | WT-6 | 0 | 0 | 0 | 0 | 0 | 0 | 0 | 0 | 0 | 0 | 0 |
|  | Sod1KO-1 | 0 | 0 | 0 | 0 | 0 | 0 | 0 | 0 | 1 | 0 | 1 |
|  | Sod1KO-2 | 0 | 1 | 0 | 0 | 0 | 0 | 0 | 0 | 2 | 0 | 3 |
|  | Sod1KO-3 | 0 | 0 | 0 | 0 | 0 | 0 | 0 | 0 | 1 | 0 | 1 |
|  | Sod1KO-4 | 0 | 1 | 0 | 0 | 0 | 0 | 0 | 0 | 2 | 0 | 3 |
|  | Sod1KO-5 | 0 | 0 | 0 | 0 | 1 | 0 | 0 | 0 | 2 | 0 | 3 |
|  | Sod1KO-6 | 0 | 0 | 0 | 0 | 0 | 0 | 0 | 0 | 1 | 0 | 1 |
|  | Sod1KO-7 | 0 | 1 | 0 | 0 | 0 | 0 | 0 | 0 | 2 | 0 | 3 |
|  | Sod1KO-8 | 0 | 1 | 1 | 0 | 1 | 0 | 0 | 0 | 1 | 0 | 4 |
|  | Sod1KO-9 | 1 | 1 | 1 | 0 | 0 | 0 | 0 | 0 | 1 | 0 | 4 |
|  | Sod1KO-10 | 0 | 1 | 0 | 0 | 1 | 0 | 0 | 2 | 0 | 0 | 4 |

**Pancreas**

| Sex | Strain | ExAtr | Pitis | Initis | Ste | LyAgg | Tum | Nod | Total |
| --- | --- | --- | --- | --- | --- | --- | --- | --- | --- |
| Male | WT-1 | 0 | 0 | 0 | 0 | 0 | 0 | 0 | 0 |
|  | WT-2 | 0 | 1 | 0 | 0 | 0 | 0 | 0 | 1 |
|  | WT-3 | 0 | 0 | 0 | 0 | 0 | 0 | 0 | 0 |
|  | WT-4 | 0 | 0 | 0 | 0 | 0 | 0 | 0 | 0 |
|  | WT-5 | 0 | 0 | 0 | 0 | 1 | 0 | 0 | 1 |
|  | WT-6 | 1 | 0 | 0 | 0 | 1 | 0 | 2 | 4 |
|  | WT-7 | 0 | 0 | 0 | 0 | 0 | 0 | 0 | 0 |
|  | WT-8 | 0 | 0 | 0 | 0 | 0 | 0 | 0 | 0 |
|  | Sod1KO-1 | 0 | 1 | 0 | 0 | 0 | 0 | 1 | 2 |
|  | Sod1KO-2 | 0 | 0 | 0 | 0 | 0 | 0 | 0 | 0 |
|  | Sod1KO-3 | 0 | 0 | 0 | 0 | 0 | 0 | 0 | 0 |
|  | Sod1KO-4 | 0 | 0 | 0 | 0 | 0 | 0 | 0 | 0 |
|  | Sod1KO-5 | 0 | 1 | 0 | 0 | 0 | 0 | 1 | 2 |
|  | Sod1KO-6 | 0 | 1 | 0 | 0 | 0 | 0 | 0 | 1 |
|  | Sod1KO-7 | 0 | 0 | 0 | 0 | 1 | 0 | 0 | 1 |
|  | Sod1KO-8 | 0 | 0 | 1 | 0 | 1 | 0 | 0 | 2 |
| Female | WT-1 | 0 | 0 | 0 | 0 | 0 | 0 | 0 | 0 |
|  | WT-2 | 0 | 0 | 0 | 0 | 1 | 0 | 0 | 1 |
|  | WT-3 | 0 | 0 | 0 | 0 | 1 | 0 | 0 | 1 |
|  | WT-4 | 0 | 0 | 0 | 0 | 0 | 0 | 0 | 0 |
|  | WT-5 | 0 | 0 | 0 | 0 | 0 | 0 | 0 | 0 |
|  | WT-6 | 0 | 0 | 0 | 0 | 0 | 0 | 0 | 0 |
|  | Sod1KO-1 | 0 | 0 | 0 | 0 | 1 | 0 | 0 | 1 |
|  | Sod1KO-2 | 0 | 0 | 1 | 0 | 1 | 0 | 0 | 2 |
|  | Sod1KO-3 | 0 | 0 | 0 | 0 | 1 | 0 | 0 | 1 |
|  | Sod1KO-4 | 0 | 0 | 0 | 0 | 1 | 0 | 0 | 1 |
|  | Sod1KO-5 | 0 | 0 | 0 | 0 | 0 | 0 | 0 | 0 |
|  | Sod1KO-6 | 0 | 0 | 0 | 0 | 1 | 0 | 0 | 1 |
|  | Sod1KO-7 | 0 | 0 | 0 | 0 | 1 | 0 | 0 | 1 |
|  | Sod1KO-8 | 0 | 0 | 0 | 0 | 1 | 0 | 0 | 1 |
|  | Sod1KO-9 | 0 | 0 | 0 | 0 | 1 | 0 | 0 | 1 |
|  | Sod1KO-10 | 0 | 0 | 1 | 0 | 0 | 0 | 0 | 1 |

**Gastrointestinal**

| Sex | Strain | Gitis | G Ulc | Enitis | C it is | Total |
| --- | --- | --- | --- | --- | --- | --- |
| Male | WT-1 | 0 | 0 | 0 | 0 | 0 |
|  | WT-2 | 0 | 0 | 0 | 0 | 0 |
|  | WT-3 | 0 | 0 | 0 | 0 | 0 |
|  | WT-4 | 0 | 0 | 0 | 0 | 0 |
|  | WT-5 | 0 | 0 | 0 | 0 | 0 |
|  | WT-6 | 0 | 0 | 0 | 0 | 0 |
|  | WT-7 | 0 | 0 | 0 | 0 | 0 |
|  | WT-8 | 0 | 0 | 0 | 0 | 0 |
|  | Sod1KO-1 | 0 | 0 | 0 | 0 | 0 |
|  | Sod1KO-2 | 0 | 0 | 0 | 0 | 0 |
|  | Sod1KO-3 | 0 | 0 | 0 | 0 | 0 |
|  | Sod1KO-4 | 0 | 0 | 0 | 0 | 0 |
|  | Sod1KO-5 | 0 | 0 | 0 | 0 | 0 |
|  | Sod1KO-6 | 0 | 0 | 0 | 0 | 0 |
|  | Sod1KO-7 | 0 | 0 | 0 | 0 | 0 |
|  | Sod1KO-8 | 0 | 0 | 0 | 0 | 0 |
| Female | WT-1 | 0 | 0 | 0 | 0 | 0 |
|  | WT-2 | 0 | 0 | 0 | 0 | 0 |
|  | WT-3 | 0 | 0 | 0 | 0 | 0 |
|  | WT-4 | 0 | 0 | 0 | 0 | 0 |
|  | WT-5 | 0 | 0 | 0 | 0 | 0 |
|  | WT-6 | 0 | 0 | 0 | 0 | 0 |
|  | Sod1KO-1 | 0 | 0 | 0 | 0 | 0 |
|  | Sod1KO-2 | 0 | 0 | 0 | 0 | 0 |
|  | Sod1KO-3 | 0 | 0 | 0 | 0 | 0 |
|  | Sod1KO-4 | 0 | 0 | 0 | 0 | 0 |
|  | Sod1KO-5 | 0 | 0 | 0 | 0 | 0 |
|  | Sod1KO-6 | 0 | 0 | 0 | 0 | 0 |
|  | Sod1KO-7 | 0 | 0 | 1 | 0 | 1 |
|  | Sod1KO-8 | 0 | 0 | 0 | 0 | 0 |
|  | Sod1KO-9 | 0 | 0 | 0 | 0 | 0 |
|  | Sod1KO-10 | 0 | 0 | 0 | 0 | 0 |

**Skin**

| Sex | Strain | it is | Min | Hyp | Total |
| --- | --- | --- | --- | --- | --- |
| Male | WT-1 | 0 | 0 | 0 | 0 |
|  | WT-2 | 0 | 0 | 0 | 0 |
|  | WT-3 | 0 | 0 | 0 | 0 |
|  | WT-4 | 0 | 0 | 0 | 0 |
|  | WT-5 | 0 | 0 | 0 | 0 |
|  | WT-6 | 0 | 0 | 0 | 0 |
|  | WT-7 | 0 | 0 | 0 | 0 |
|  | WT-8 | 0 | 0 | 0 | 0 |
|  | Sod1KO-1 | 0 | 0 | 0 | 0 |
|  | Sod1KO-2 | 0 | 0 | 0 | 0 |
|  | Sod1KO-3 | 0 | 0 | 1 | 1 |
|  | Sod1KO-4 | 0 | 0 | 0 | 0 |
|  | Sod1KO-5 | 0 | 0 | 0 | 0 |
|  | Sod1KO-6 | 0 | 0 | 0 | 0 |
|  | Sod1KO-7 | 0 | 0 | 0 | 0 |
|  | Sod1KO-8 | 0 | 0 | 0 | 0 |
| Female | WT-1 | 0 | 0 | 0 | 0 |
|  | WT-2 | 0 | 0 | 0 | 0 |
|  | WT-3 | 0 | 0 | 0 | 0 |
|  | WT-4 | 0 | 0 | 0 | 0 |
|  | WT-5 | 0 | 0 | 0 | 0 |
|  | WT-6 | 0 | 0 | 0 | 0 |
|  | Sod1KO-1 | 0 | 0 | 0 | 0 |
|  | Sod1KO-2 | 0 | 0 | 0 | 0 |
|  | Sod1KO-3 | 0 | 0 | 0 | 0 |
|  | Sod1KO-4 | 0 | 0 | 0 | 0 |
|  | Sod1KO-5 | 0 | 0 | 0 | 0 |
|  | Sod1KO-6 | 0 | 0 | 0 | 0 |
|  | Sod1KO-7 | 0 | 0 | 0 | 0 |
|  | Sod1KO-8 | 0 | 0 | 0 | 0 |
|  | Sod1KO-9 | 0 | 0 | 0 | 0 |
|  | Sod1KO-10 | 0 | 0 | 0 | 0 |

**Male and Female Reproductive systems**

**Male Female**

| Sex | Strain | T Deg | Oritis | Arteritis | AGG Ect | AGG it is | Total | CEM | It is | Ovary | Tot |
| --- | --- | --- | --- | --- | --- | --- | --- | --- | --- | --- | --- |
| Male | WT-1 | 0 | 0 | 0 | 1 | 0 | 1 | NP | NP | NP | 0 |
|  | WT-2 | 0 | 0 | 0 | 1 | 0 | 1 | NP | NP | NP | 0 |
|  | WT-3 | 0 | 0 | 0 | 1 | 0 | 1 | NP | NP | NP | 0 |
|  | WT-4 | 0 | 0 | 0 | 1 | 0 | 1 | NP | NP | NP | 0 |
|  | WT-5 | 0 | 0 | 0 | 0 | 0 | 0 | NP | NP | NP | 0 |
|  | WT-6 | 0 | 0 | 0 | 0 | 0 | 0 | NP | NP | NP | 0 |
|  | WT-7 | 0 | 0 | 0 | 0 | 0 | 0 | NP | NP | NP | 0 |
|  | WT-8 | 0 | 0 | 0 | 0 | 0 | 0 | NP | NP | NP | 0 |
|  | Sod1KO-1 | 1 | 0 | 0 | 1 | 0 | 2 | NP | NP | NP | 0 |
|  | Sod1KO-2 | 3 | 0 | 0 | 1 | 0 | 4 | NP | NP | NP | 0 |
|  | Sod1KO-3 | 1 | 0 | 0 | 1 | 0 | 2 | NP | NP | NP | 0 |
|  | Sod1KO-4 | 1 | 0 | 0 | 1 | 0 | 2 | NP | NP | NP | 0 |
|  | Sod1KO-5 | 2 | 0 | 0 | 1 | 0 | 3 | NP | NP | NP | 0 |
|  | Sod1KO-6 | 1 | 0 | 0 | 1 | 0 | 2 | NP | NP | NP | 0 |
|  | Sod1KO-7 | 1 | 0 | 0 | 0 | 1 | 2 | NP | NP | NP | 0 |
|  | Sod1KO-8 | 1 | 0 | 0 | 0 | 1 | 2 | NP | NP | NP | 0 |
| Female | WT-1 | NP | NP | NP | NP | NP | 0 | 2 | 0 | 0 | 2 |
|  | WT-2 | NP | NP | NP | NP | NP | 0 | 1 | 0 | 0 | 1 |
|  | WT-3 | NP | NP | NP | NP | NP | 0 | 2 | 1 | 0 | 3 |
|  | WT-4 | NP | NP | NP | NP | NP | 0 | 2 | 0 | 0 | 2 |
|  | WT-5 | NP | NP | NP | NP | NP | 0 | 3 | 0 | 0 | 3 |
|  | WT-6 | NP | NP | NP | NP | NP | 0 | 2 | 0 | 0 | 2 |
|  | Sod1KO-1 | NP | NP | NP | NP | NP | 0 | 2 | 1 | 0 | 3 |
|  | Sod1KO-2 | NP | NP | NP | NP | NP | 0 | 2 | 2 | 0 | 4 |
|  | Sod1KO-3 | NP | NP | NP | NP | NP | 0 | 1 | 0 | 0 | 1 |
|  | Sod1KO-4 | NP | NP | NP | NP | NP | 0 | 1 | 0 | 0 | 1 |
|  | Sod1KO-5 | NP | NP | NP | NP | NP | 0 | 3 | 0 | 0 | 3 |
|  | Sod1KO-6 | NP | NP | NP | NP | NP | 0 | 1 | 0 | 0 | 1 |
|  | Sod1KO-7 | NP | NP | NP | NP | NP | 0 | 2 | 1 | 0 | 3 |
|  | Sod1KO-8 | NP | NP | NP | NP | NP | 0 | 2 | 0 | 0 | 2 |
|  | Sod1KO-9 | NP | NP | NP | NP | NP | 0 | 2 | 0 | 0 | 2 |
|  | Sod1KO-10 | NP | NP | NP | NP | NP | 0 | 1 | 0 | 0 | 1 |

**Salivary Gland**

| Sex | Strain | Atr | IT IS | LyAgg | Total |
| --- | --- | --- | --- | --- | --- |
| Male | WT-1 | 0 | 0 | 0 | 0 |
|  | WT-2 | 0 | 0 | 0 | 0 |
|  | WT-3 | 0 | 0 | 0 | 0 |
|  | WT-4 | 0 | 1 | 0 | 1 |
|  | WT-5 | 0 | 0 | 0 | 0 |
|  | WT-6 | NP | NP | NP | 0 |
|  | WT-7 | 0 | 1 | 0 | 1 |
|  | WT-8 | 0 | 1 | 0 | 1 |
|  | Sod1KO-1 | 0 | 1 | 1 | 2 |
|  | Sod1KO-2 | 0 | 1 | 1 | 2 |
|  | Sod1KO-3 | 0 | 0 | 0 | 0 |
|  | Sod1KO-4 | 0 | 1 | 0 | 1 |
|  | Sod1KO-5 | 0 | 1 | 1 | 2 |
|  | Sod1KO-6 | 0 | 1 | 0 | 1 |
|  | Sod1KO-7 | 0 | 0 | 0 | 0 |
|  | Sod1KO-8 | 0 | 1 | 2 | 3 |
| Female | WT-1 | 0 | 1 | 1 | 2 |
|  | WT-2 | 0 | 0 | 1 | 1 |
|  | WT-3 | 0 | 0 | 1 | 1 |
|  | WT-4 | 0 | 0 | 1 | 1 |
|  | WT-5 | 0 | 0 | 0 | 0 |
|  | WT-6 | 0 | 0 | 1 | 1 |
|  | Sod1KO-1 | 0 | 0 | 1 | 1 |
|  | Sod1KO-2 | 0 | 1 | 1 | 2 |
|  | Sod1KO-3 | 0 | 0 | 1 | 1 |
|  | Sod1KO-4 | 0 | 0 | 2 | 2 |
|  | Sod1KO-5 | 0 | 0 | 2 | 2 |
|  | Sod1KO-6 | 0 | 0 | 1 | 1 |
|  | Sod1KO-7 | 0 | 0 | 2 | 2 |
|  | Sod1KO-8 | 0 | 0 | 1 | 1 |
|  | Sod1KO-9 | 0 | 1 | 1 | 2 |
|  | Sod1KO-10 | 1 | 0 | 0 | 1 |

**Neuro**

| Sex | Strain | Sp Cord |
| --- | --- | --- |
| Male | WT-1 | 0 |
|  | WT-2 | 0 |
|  | WT-3 | 0 |
|  | WT-4 | 0 |
|  | WT-5 | 0 |
|  | WT-6 | 0 |
|  | WT-7 | 0 |
|  | WT-8 | 0 |
|  | Sod1KO-1 | 0 |
|  | Sod1KO-2 | 0 |
|  | Sod1KO-3 | 0 |
|  | Sod1KO-4 | 0 |
|  | Sod1KO-5 | 0 |
|  | Sod1KO-6 | 0 |
|  | Sod1KO-7 | 0 |
|  | Sod1KO-8 | 0 |
| Female | WT-1 | 0 |
|  | WT-2 | NP |
|  | WT-3 | 0 |
|  | WT-4 | 0 |
|  | WT-5 | 0 |
|  | WT-6 | 0 |
|  | Sod1KO-1 | 0 |
|  | Sod1KO-2 | 0 |
|  | Sod1KO-3 | 0 |
|  | Sod1KO-4 | 0 |
|  | Sod1KO-5 | 0 |
|  | Sod1KO-6 | 0 |
|  | Sod1KO-7 | 0 |
|  | Sod1KO-8 | 0 |
|  | Sod1KO-9 | 0 |
|  | Sod1KO-10 | 0 |
